# Respiratory influence on left atrial volume calculation with 3D-echocardiography

**DOI:** 10.1186/s12947-016-0054-7

**Published:** 2016-03-12

**Authors:** Mathias Sørgaard, Jesper J. Linde, Hafsa Ismail, Niels Risum, Klaus F. Kofoed, Jørgen T. Kühl, Benjamin Tittle, Walter B. Nielsen, Jens D. Hove

**Affiliations:** 1 Department of Cardiology, The Heart Centre, Rigshospitalet, University of Copenhagen, Blegdamsvej 9, 2100-CPH København, Denmark; 2 Department of Cardiology, Hvidovre Hospital, University of Copenhagen, København, Denmark; 3 Department of Radiology, Rigshospitalet, University of Copenhagen, København, Denmark; 4 Centre for Functional and Diagnostic Imaging and Research, Hvidovre Hospital, University of Copenhagen, København, Denmark

**Keywords:** Three dimensional transthoracic echocardiography, Left atrial volume, Quantitation, Respiration

## Abstract

**Background:**

Left atrial volume (LAV) estimation with 3D echocardiography has been shown to be more accurate than 2D volume calculation. However, little is known about the possible effect of respiratory movements on the accuracy of the measurement.

**Methods:**

100 consecutive patients admitted with chest pain were examined with 3D echocardiography and LAV was quantified during inspiratory breath hold, expiratory breath hold and during free breathing.

**Results:**

Of the 100 patients, only 65 had an echocardiographic window that allowed for 3D echocardiography in the entire respiratory cycle. Mean atrial end diastolic volume was 45.4 ± 14.5 during inspiratory breath hold, 46.4 ± 14.8 during expiratory breath hold and 45.6 ± 14.3 during free respiration. Mean end systolic volume was 17.6 ± 7.8 during inspiratory breath hold, 18.8 ± 8.0 during expiratory breath hold and 18.3 ± 8.0 during free respiration. No significant differences were seen in any of the measured parameters.

**Conclusions:**

The present study adds to the feasibility of 3D LAV quantitation. LAV estimation by 3D echocardiography may be performed during either end-expiratory or end-inspiratory breath-hold without any significant difference in the calculated volume. Also, the LAV estimation may be performed during free breathing.

## Background

Left atrial (LA) size and function has emerged as a clinically useful parameter in predicting atrial fibrillation [1–3], risk of stroke [4, 5], heart failure [6, 7], and death [4, 8]. Also, LA volume (LAV) is related to the severity of left ventricular diastolic dysfunction [9, 10]. Two-dimensional (2D) echocardiography has been used to assess LAV [11, 12] but it has been shown to significantly underestimate the size of the LA compared to magnetic resonance imaging (MRI) and computer tomography (CT) [13, 14]. Three dimensional (3D) echocardiography has evolved during the last decade, and the use of multidimensional phase-array detectors have allowed for real time 3D-echocardiography to be performed in a clinical setting. A major advantage of this technique is the independency of geometric assumptions. A number of studies have demonstrated the feasibility of 3D echocardiography for the assessment of LAV, and it has been validated against magnetic resonance imaging (MRI) [15–17], and multidetector computed tomography (MDCT) [18, 19].

Image quality is crucial for accurate diagnosis and quantitation. Conventional image acquisition for LAV assessment is performed during breath hold [20]. In some patients a better echo window may be present at end-inspiration, while other patients with pulmonary disease may have difficulties in cooperating.

Studies suggest that respiratory variations may affect the LAV. Several studies have shown a decrease in the left atrial size in healthy volunteers following increased intrathoracic pressure [21–23]. However, it is unknown whether normal respiratory variation interfere with LAV measurement and whether this should be accounted for during image acquisition. Accordingly, the current study was designed to study the possible influence of respiratory variations on 3D echocardiographic assessment of LAV and function and examine the influence of free breathing.

## Methods

### Study patients

Patients for this study were derived from the cohort of patients included in the CATCH trial (CArdiac cT in the treatment of acute CHest pain) [24]. With approval from the institutional review board and the local ethics committee, we prospectively enrolled 100 patients with suspected coronary artery disease and performed 3D transthoracic echocardiography for evaluation of cardiac function. Informed consent was obtained from all enrolled patients. The study was performed in accordance with the declaration of Helsinki.

### Echocardiographic data acquisition and LAV by Real-Time 3D echocardiography

Transthoracic echocardiography was performed by an experienced cardiologist (TTE and TEE certified by the European Society of Cardiology) with patients in the left lateral decubitus position using a commercially available echocardiographic system (iE33; Philips Medical Systems, Andover, MA). All echocardiographic images were stored digitally, and measurements were performed offline using the commercially available QLAB software package (Philips Medical Systems). Measurements were obtained for three consecutive beats and averaged for all calculations. LAV was indexed to body surface area. Image quality was visually divided into 5 groups, according to the following classification. 5 = optimal image quality, 4 = slightly reduced image quality, without implications on interpretation, 3 = reduced image quality with few interpretation difficulties, 2 = reduced image quality with extensive interpretation difficulties, 1 = poor image quality with severe implications on interpretation or non-diagnostic image quality.

**Fig. 1:**
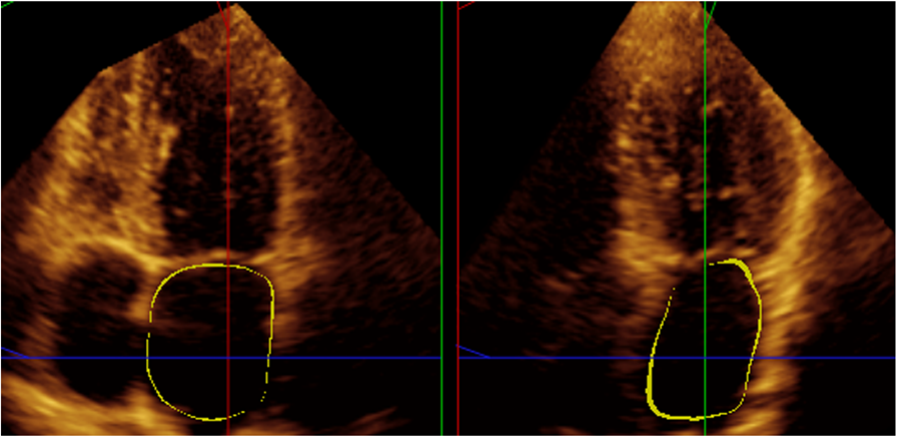
The QLAB echo image from the analysis of a patient is shown. The image illustrates the border delineation during atrial diastole. LAV was measured from endocardial tracing. At the valve leaflets the tracing was limited to the annular plane

LAV by real-time 3D echocardiography was collected in full-volume mode during inspiratory breath hold, expiratory breath hold and free breathing, by instruction from the operator, using an ×3-1 matrix-array transducer. The correct breath holding was verified by visual inspection before the subsequent data acquisition. Acquisition was triggered to the electrocardiographic R wave. Care was taken to ensure that the entire LA was included within a pyramidal 3D data set. Image acquisition was further optimized to ensure the highest possible frame rate (adjustment of sector width and depth). LAV by 3D echocardiography was derived from semiautomatic tracing of the LA endocardium, at ventricular end-systole in the apical 4- and 2 chamber planes, using Philips QLAB software. This was performed by marking 5 points in the atrial surfaces of the mitral annulus: at the anterior, inferior, lateral, and septal annuli, and the fifth point at the apex of the left atrium. Once this was complete, the endocardial surface was automatically delineated, and a mathematical model of the left atrium could be visualized from different points of views, and the LAV calculation was obtained (Fig. 1). Manual modification was made to correct the automatic tracings when necessary. At the valve leaflets the tracing was limited to the annular plane.

We measured the LAV, at end atrial diastole and systole. Atrial diastole and systole was determined during the analyses by visual inspections of the time points for mitral valve opening and closing. From these volumes the LA ejection fraction and LA stroke volume were calculated. The volumes were quantified during inspiratory breath hold, expiratory breath hold and during free breathing. All the inspiratory images were analyzed first, then the expiratory images, and in the end the images during free breathing. This was to ensure, that previous results from the same patient, would not be memorable for the person doing the analyses.

Inter- and intra-observatory analyses were made on 20 randomly selected studies, at free breathing, to make sure that results were reproducible.

### Statistical analysis

Statistical analyses were performed using SAS version 9.2. Continuous variables were described as means ± SD and compared with paired *t* tests. Bland-Altman plots were used to compare the different methods and were constructed using GraphPad Prism 6, CA 92037, USA.

## Results

Patient characteristics are shown in Table 1. 100 patients were included but 35 had insufficient echo windows that did not allow for 3D echocardiography independently of respiration (18 due to poor overall image quality in 17 patients a satisfactory echo window was not obtainable at both inspiratory and expiratory breath-hold). No significant difference was seen in the characteristics between the patients that were included and those that were excluded.

**Table 1:** Study population demographics

Patients (N)	65
Male gender–no. (%):	43 (66)
*Age*–*years*	
Mean	52.9
Range	22-82
*BMI*	
Mean	26.3
Range	18-37
*Coronary artery disease Risk factors*–*no* (%)	
Hypertension	23 (35.4)
Hypercholesterolemia	24 (36.9)
Family history of CAD	14 (21.5)
Diabetes Mellitus	4 (6.2)
Tobacco use	
Current smokers	25 (38.5)
Ever smokers	43 (66.2)
Pack years mean	21.9
Previous myocardial infarction	9 (13.8)
Known ischemic heart disease	11 (16.9)
Previous apoplexies/TCI (%)	4 (6.2)
Pheripheral vascular disease (%)	1 (1.5)
LVEF (range)	58.8 (25–60)

*BMI* body mass index, *CAD* coronary artery disease, *TCI* transitory cerebral ischemia

The studies had an average quality of 3.5, so quality above 3.5 was classified as high, and quality below 3.5 were classified as low. Using this classification, 40 patients had high quality images, and 25 had low quality images. The mean frame rate was 16 ± 5 frames/sec.

The LA size was normal in 58 patients, mildly dilated in 2 patients, moderately dilated in 3 patients and severely dilated in 2 patients according to normal reference values of the size of the left atrium [25].

### End diastolic atrial volume

**Fig. 2:**
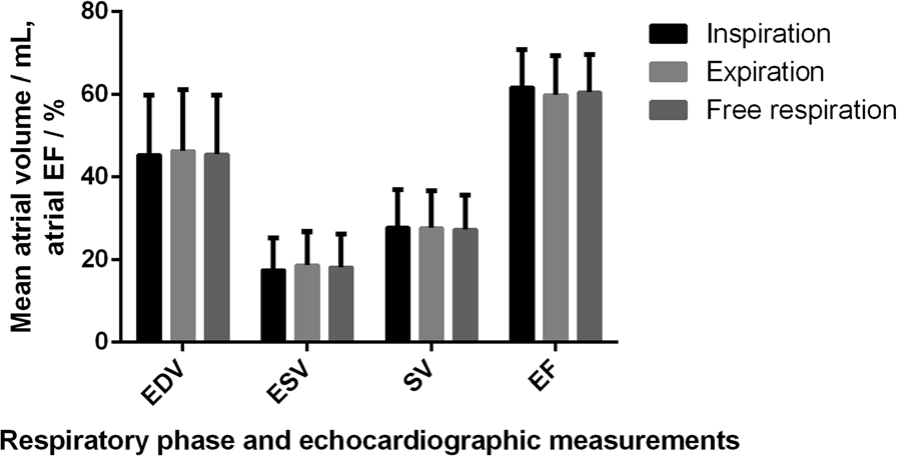
Mean end diastolic volume, end systolic volume, stroke volume and ejection fraction of the left atrium, during the different respiratory phases. Volumes are shown as mL’s and EF as %. Vertical lines are showing the standard deviations. No significant differences were found. EDV = end diastolic atrial volume, ESV = end systolic atrial volume, SV = atrial stroke volume, EF = atrial ejection fraction

Mean end diastolic atrial volume (EDV) was 45.4 mL ± 14.5 mL during inspiratory breath hold, 46.4 mL ± 14.8 mL during expiratory breath hold and 45.6 mL ± 14.3 mL during normal respiration (Fig. 2).

### End systolic atrial volume

Mean end systolic atrial volume (ESV) was 17.6 mL ± 7.8 mL during inspiratory breath hold, 18.8 mL ± 8.0 mL during expiratory breath hold and 18.3 mL ± 8.0 mL during normal respiration (Fig. 2). (inspiratory vs. expiratory, *p* = 0.11; inspiratory vs. normal, *p* = 0.21; expiratory vs. normal, *p* = 0.63).

### Atrial stroke volume

Mean atrial stroke volume (SV) was 27.8 mL ± 9.1 mL during inspiratory breath hold, 27.6 mL ± 9.0 mL during expiratory breath hold and 27.3 mL ± 8.3 mL during normal respiration (Fig. 2) (inspiratory vs. expiratory, *p* = 0.86; inspiratory vs. normal, *p* = 0.62; expiratory vs. normal, *p* = 0.74).

### Atrial ejection fraction

Mean atrial ejection fraction (EF) was 61.8 % ± 9.1 % during inspiratory breath hold, 60.0 % ± 9.3 % during expiratory breath hold and 60.6 % ± 9.0 % during normal respiration (Fig. 2) (inspiratory vs. expiratory, *p* = 0.23; inspiratory vs. normal, *p* = 0.37; expiratory vs. normal, *p* = 0.63).

**Fig. 3:**
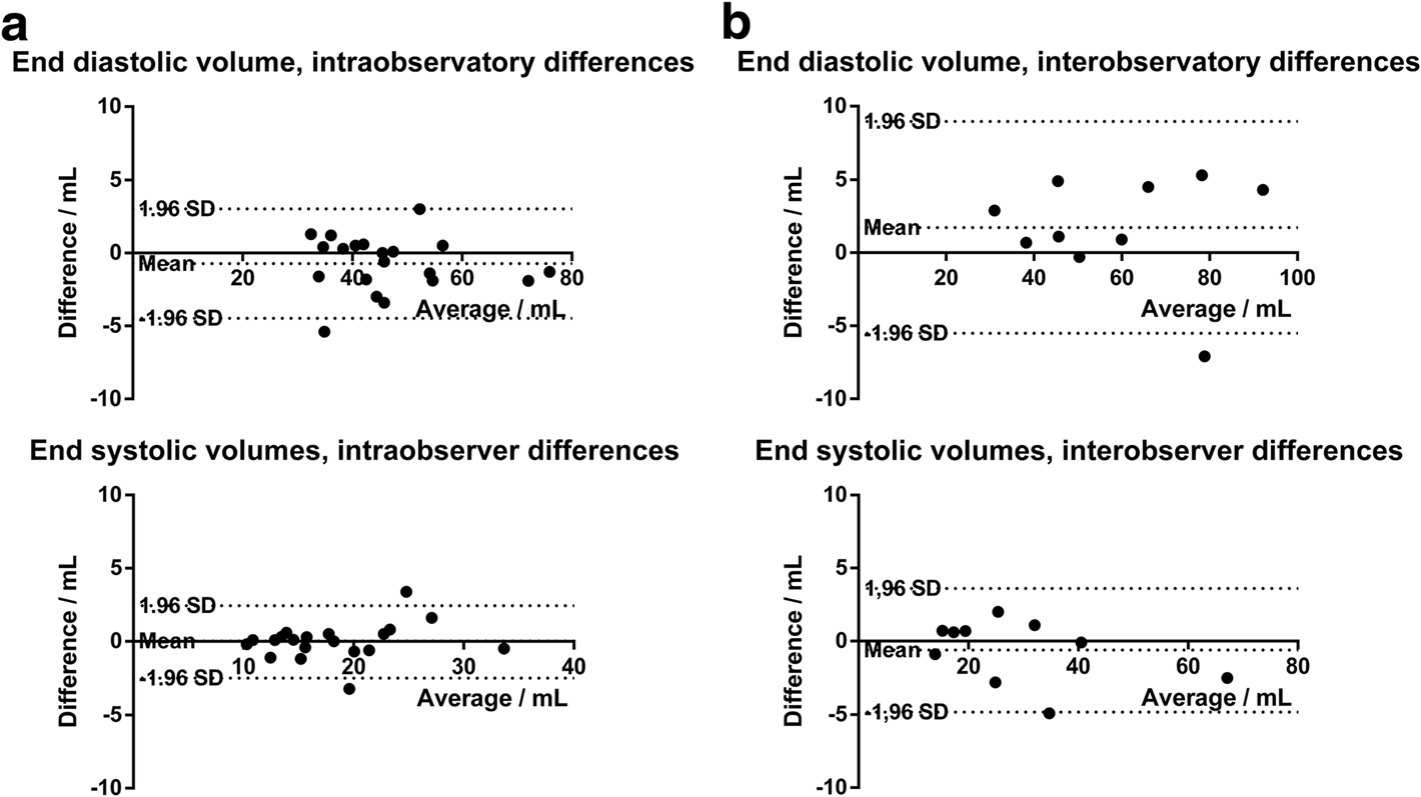
**a** Bland-Altman plot of the intraobserver end diastolic and systolic atrial volume measurements during free breathing, showing a small intraobserver difference. **b** Bland-Altman plot of the interobserver end diastolic and systolic atrial volume measurements during free breathing, showing a small interobserver difference

There was no significant intra – or interobserver differences between any of the measured parameters (Fig. 3).

**Fig. 4:**
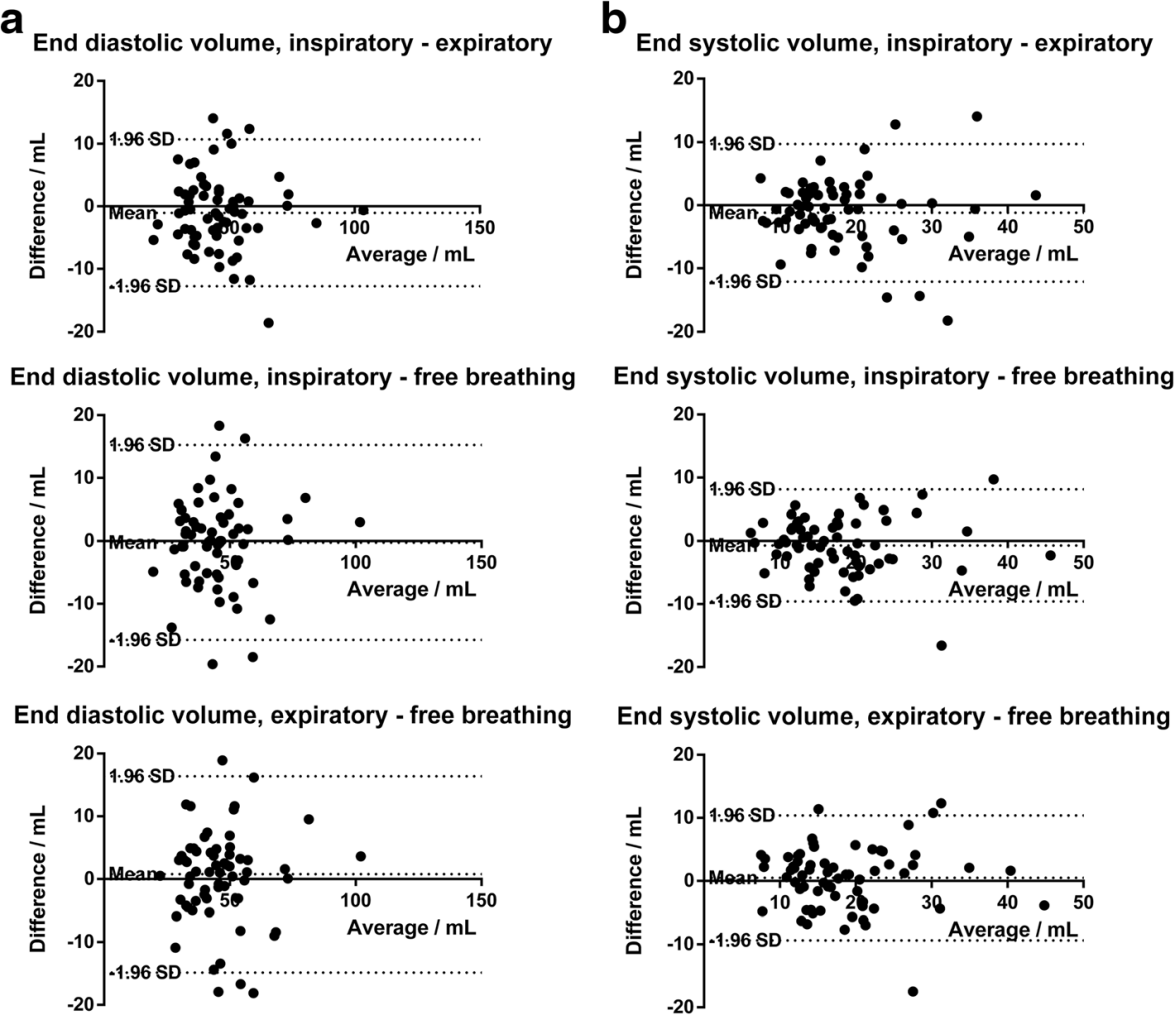
**a** Bland-Altman plot for end diastolic volumes showing differences between the measurements at the various breathing phases (Inspiratory vs. expiratory: bias = 1.0 mL ± 6.0 mL, inspiratory vs. free breathing: bias = −0.3 mL ± 7.9 mL and expiratory vs. free breathing: bias = 0.8 mL ± 8.0 mL). **b** Bland-Altman plot for end systolic volumes showing differences between the measurements at the various breathing phases (Inspiratory vs. expiratory: bias = −1.2 mL ± 5.6 mL, inspiratory vs. free breathing: bias = −0.7 mL ± 4.5 mL and expiratory vs. free breathing: bias = 0.5 mL ± 5.0 mL)

There were no respiratory differences at neither high nor low atrial volumes. This is shown by Bland-Altman plots (Fig. 4a and b), where points are distributed equally around the mean, at high, low and intermediate volumes. Further statistical results on the differences of the various measurements and respiratory phases are shown in Table 2. Furthermore, when dividing the population with regard to image quality, no significant interobserver or intraobserver differences were seen between the measured parameters in the low-quality group versus in the high-quality group.

**Table 2:** Statistics on the differences between the various measurements and respiratory states

		Mean bias ± SD	Correlation coefficiency	95 % limits of agreement	Coefficient of variation
EDV	Inspiratory vs. expiratory	−1.0 ± 6.0	−0.043 (*p* = 0.73)	−12.79-10.71	9.3 %
	Inspiratory vs. free breathing	−0.26 ± 7.9	0.038 (*p* = 0.77)	−15.75-15.24	12.2 %
	Expiratory vs. free breathing	0.78 ± 8.0	0.071 (*p* = 0.58)	−14.83-16.40	12.2 %
ESV	Inspiratory vs. expiratory	−1.19 ± 5.6	−0.048 (*p* = 0.71)	−12.11-9.72	19.1 %
	Inspiratory vs. free breathing	−0.70 ± 4.5	−0.04 (*p* = 0.72)	−9.57-8.16	15.9 %
	Expiratory vs. free breathing	0.49 ± 5.0	0.011 (*p* = 0.93)	−9.39-10.37	17.4 %
SV	Inspiratory vs. expiratory	0.16 ± 7.2	0.011 (*p* = 0.93)	−13.91-14.23	18.2 %
	Inspiratory vs. free breathing	0.48 ± 7.8	0.12 (*p* = 0.33)	−14.86-15.82	20.0 %
	Expiratory vs. free breathing	0.31 ± 7.5	0.12 (*p* = 0.36)	−14.38-15.01	19.2 %
EF	Inspiratory vs. expiratory	1.77 ± 11.8	−0.027 (*p* = 0.83)	−21.41-24.95	13.8 %
	Inspiratory vs. free breathing	1.13 ± 10.0	0.013 (*p* = 0.92)	−18.53-20.78	11.6
	Expiratory vs. free breathing	−0.64 ± 10.8	0.040 (*p* = 0.75)	−21.80-20.52	12.6 %

*EF* atrial ejection fraction, *EDV* end diastolic atrial volume, *ESV* end systolic atrial volume, *SD* standard deviation, *SV* atrial stroke volume

## Discussion

LAV provides information on the severity of left ventricular diastolic dysfunction, and has also been shown to carry important prognostic information. In addition, LAV by echocardiography and MDCT has been shown to be a predictor of death in patients with acute myocardial infarction [26, 27]. Hence, LAV is an important measurement in a large subset of patients with heart disease, and hence it is important that the 3D image acquisition is as convenient as possible.

The present study investigates for the first time the influence of respiration on the LAV assessment by 3D echocardiography. The study shows that the LA quantitative parameters are not significantly affected by respiration. Thus LAV can be acquired accurately from image acquisition during end-expiration, end-inspiration or free breathing.

In this population 35 patients out of 100 were excluded because the image quality was insufficient for measuring the LAV by 3D echocardiography in all respiratory states. Our results show that the LAV may be measured in any respiratory state, and since only 18 patients were excluded because of poor overall image quality, the amount of patients in whom 3D echocardiographic measurement of the LAV is not possible may be reduced significantly, by selecting the respiratory state with the best view of the LA.

Early studies of LAV during mechanical ventilation indicated, that positive end-expiratory pressure might influence the LAV. Leithner et al. showed a progressive decrease in the LA size, in healthy volunteers when the PEEP pressure increased from 7 to 15 cm water using cardiac MR [23]. The volumes decreased in relation to simultaneous increase in lung volumes, and it was hypothesized that the reduced cardiac volumes were caused by cardiac compression related to the lung expansion. The volume decline during PEEP ventilation is supported by Riddervold et al., who showed a volume decline using sonomicrometry in an open and close chest dog experiment [21]. Presumably, lung expansion during end-inspiration did not reach the same extent, in this study. Also, the lung volume expansion in our study is more physiologic and may be associated with potential counteracting effects. Thus, during inspiration the right heart volume increases (due to negative intra thoracic pressure), whereas it decreases during PEEP pressure.

### Limitations

Most of the patients had normal ejection fraction and a structurally normal heart. Cardiac diseases affecting the ejection fraction (constrictive pericarditis), or causing increased sensibility to intrathoracic pressure changes during respiration, are not accounted for in this study. Furthermore, we did not examine any patients suffering from serious respiratory distress. Other studies are necessary to explore these topics. Our patients, however, are representative of cardiac patients with a fairly high body-mass index affecting image quality. Image quality per se, however, did not seem to have any effect on the LAV quantification in either respiration cycle.

## Conclusion

Our study indicates that differences in LAV related to respiration are negligible during normal breathing. In addition, we find that free breathing does not have influence on the LAV quantification.
